# miR-618: possible control over TIMP-1 and its expression in localized prostate cancer

**DOI:** 10.1186/s12885-018-4930-4

**Published:** 2018-10-19

**Authors:** Renato F. Ivanovic, Nayara I. Viana, Denis R. Morais, Caio Moura, Iran A. Silva, Katia R. Leite, José Pontes-Junior, William C. Nahas, Miguel Srougi, Sabrina T. Reis

**Affiliations:** 1 Laboratory of Medical Investigation (LIM55), Urology Department, University of Sao Paulo Medical School, Av. Dr. Arnaldo 455, 2nd floor, room 2145, Sao Paulo, 01246-903 Brazil; 2 Uro-Oncology Group, Urology Department, University of Sao Paulo Medical School and Institute of Cancer Estate of Sao Paulo (ICESP), Sao Paulo, Brazil

**Keywords:** Prostate cancer, MMP-9, TIMP-1, microRNA, Invasion

## Abstract

**Background:**

The imbalance between the action of the tissue inhibitors of matrix metalloproteinases (TIMPs) and the matrix metalloproteinases (MMPs) is one component of metastasis physiology. TIMP-1 overrides MMP-9 activity in cancer and might be regulated by miR-618. The aims of this study were to clarify whether TIMP-1 expression is modified by miR-618 and to clarify the effect of miR-618 expression on the invasion of prostate cancer cells. We also studied miR-618 expression in surgical specimens of patients with localized prostate cancer submitted to open radical prostatectomy.

**Methods:**

After transfection of miR-618 or its antagonist in DU145 cells, qRT-PCR for TIMP-1/MMP-9 and both ELISA and zymography for MMP-9 were performed. Total miRNA was extracted from surgical specimens of PCa, and miR-618 expression was examined for correlations with Gleason score, pathological status and biochemical recurrence.

**Results:**

DU145 cells transfected with miR-618 had a 76% reduction in TIMP-1 expression relative to control cells (*p* = 0.003). miR-618 inhibition reduced MMP-9 expression by 31% (*p* = 0.032) and MMP-9 absorbance evaluated with ELISA assay (*p* = 0.06).Zymography suggested higher MMP-9 activity in DU145 cells transfected with miR-618 than those transfected with miR-618 inhibitor, but the difference was not significant (*p* = 0.55). However, miR-618 expression was lower in surgical specimens of patients with Gleason score > 7 (*p* = 0.08) and more advanced disease (*p* = 0.07).

**Conclusions:**

In vitro, miR-618 overexpression decreases TIMP-1 and miR-618 inhibition decreases MMP-9, suggesting that miR-618 might be an oncomiR. However, the analysis of clinical samples of localized prostate cancer revealed an inconsistent pattern, as increased miR-618 expression was associated with lower Gleason score and pathological status. Further studies are needed to address whether miR-618 is a context-dependent miRNA.

## Background

As new tumor markers for prostate cancer (PCa) are discovered, their usefulness for PCa detection, diagnosis, staging and prognosis are increasingly described in the medical literature [1–3].

Among the promising molecular markers for PCa are the genes belonging to the family of matrix metalloproteinases (MMPs), which is a group of proteolytic enzymes responsible for extracellular matrix degradation. The activity of MMPs is under control of the tissue inhibitors of MMP (TIMPs), and studies show that TIMPs can regulate MMPs in neoplastic diseases, including PCa [4, 5]. However, TIMPs can be controlled by a class of molecules known as microRNAs, which are composed of 19–25 nucleotides and regulate many physiological and pathological processes [6].

In cancer, an imbalance between MMPs and TIMPs leads to an excess of degradative activity, and this imbalance contributes to the invasive behavior of tumor cells. In PCa, MMP-9 has been reported to be regulated by different miRs, although studies addressing whether TIMP-1 is also subjected to the same level of control are lacking. TIMP-1 has a complementary sequence at the 3’-UTR end that may be a binding site for miR-618. This miRNA has been shown to modulate metastasis in prostate cancer cell lines through the FOXP2 gene but not through TIMP-1 [7]. Thus, we performed an in vitro study to clarify the effect of miR-618 transfection on TIMP-1 and MMP-9 expression. We also analyzed surgical specimens of PCa to identify the patterns of miR-618 expression across different Gleason scores and pathological stages.

## Methods

### MicroRNAs

miR-618 may be a regulator of TIMP-1 molecule according to target prediction tools (http://www.targetscan.org). mir-618, anti-miR-618 and positive and negative controls (Ambion, Austin, TX, USA) were diluted to 10 μM stock solutions and stored frozen at − 20 °C until use. All experiments were performed in triplicate.

### Cell lines

The DU145 cell line was used (American Type Culture Collection - ATCC). Cells were placed in medium containing DMEM supplemented with 10% fetal bovine serum (FBS) and 1% antibiotic/antimycotic solution (Sigma Co., St. Louis, MO, USA). The plates were maintained at 37 °C, 95% air and 5% CO2.

### Cell transfection

Transfections were performed with Lipofectamine (siPORT NeoFX -AMBION, USA) with the following protocol: The day before transfection, 6 × 10^4^ cells were maintained without antibiotic. Approximately 2.5 μL of 10 μM solution was diluted in 50 mL of OPTI-MEM and mixed with a solution of 1.5 μL of transfection agent diluted in 50 mL of OPTI-MEM I. Then, 100 μL of transfection complex was dispensed on a 12-well culture plate and incubated for 24 h in CO2 at 37 °C.

### Total RNA and miRNA extraction

Twenty-four hours after transfection, the cells were trypsinized and centrifuged at 4000 rpm for 5 min. Total RNA and miRNA were extracted with a mirVana kit (Applied Biosystems, Foster City, CA, USA). The purity and concentration of the miRNA and RNA were measured with a spectrophotometer (ND-1000, Thermo Scientific, Wilmington, USA) at a wavelength between 260 and 280 nm (A260/280).

### Reverse transcription (RT)

Reverse transcription was performed using the TaqMan Reverse Transcription kit (Applied Biosystems) according to the manufacturer’s instructions. The synthesis of TIMP-1 cDNA was performed with 5 ng of mRNA (High-Capacity cDNA Reverse Transcription Kit - Applied Biosystems) using reverse transcriptase and random primers.

For miRNA, the reaction was performed with Veriti equipment (Applied Biosystems) with the following parameters: 30 min at 16 °C, 30 min at 42 °C and 5 min at 85 °C. The cDNA from RNA was obtained with same equipment using the following parameters: 10 min at 25 °C, 120 min at 37 °C and 5 min at 85 °C.

### Real-time PCR

We used the ABI 7500 FAST thermocycler to assess the efficacy of transfection and the expression of the TIMP-1 and MMP-9 genes. The reactions were performed with 0.5 μL of specific primer, 5 μL of TaqMan® Universal PCR Master Mix (Applied Biosystems, California, USA), 3.5 μL of nuclease-free water and 1 μL of RNA cDNA. Primers employed in the study detected mature miRNAs. The PCR cycles were 2 min at 50 °C, 10 min at 95 °C, 40 cycles of 15 s 95 °C and 1 min at 60 °C. The endogenous control was B2M for the genes and RNU43 for the miRNAs. The expression levels of the miRNAs and target genes were determined, and the relative quantification of expression levels, expressed in fold changes, was determined by the 2^-ΔΔct^ method [8].

### Elisa

The ELISA (R&D Systems, Minneapolis, MN, USA) assay for MMP-9 detection was conducted with the conditioned medium from DU145 cells transfected with miR-618 or its antagonist according to the manufacturer’s instructions. A standard curve was prepared on each plate in duplicate. Samples were diluted two-fold for analysis. All analyses were performed in duplicate, and positive and negative controls were employed for statistical analysis.

### Zymography

The conditioned medium from the top of the Matrigel membrane was collected, and total protein was quantified using a BCA Protein Assay kit (Thermo Scientific). Approximately 40 μL of sample was added to 4–10% polyacrylamide gel containing 0.1% gelatin. Ten microliters of the protein standard (Chemicon Co.) was also loaded onto each gel. Electrophoresis was then performed at 4 °C, and the gel was washed in Tris including 2.5% Triton X-100 (Bio-Rad#161–0407) to remove the SDS and allow protein renaturation. Then, the gels were incubated for 48 h in buffer solution gel (BIO-RAD 10x Zymogram #161–0766) and stained with Coomassie blue R-250. Revelation was performed after washing the gels with a solution of 40% methanol and 10% glacial acetic acid in distilled water. MMP-9 activity was inferred at the 72 kDa band and quantified with ImageJ (Wayne Rasband, NIH, USA). Each zymography experiment was performed in triplicate.

### PCa specimens

Soon after the prostate was removed from the patient, we collected a sample of tissue from an area that we believed based on appearance to be compromised by cancer. An adjacent section of this region was sent for histological analysis, and a pathologist expert in urologic malignancies confirmed the presence of neoplasia in 75% of the samples (K.R.L). Frozen prostate-tissue samples were placed in 1.5-mL microtubes, each containing 500 mL of lysis buffer from the mirVana miRNA isolation kit (Ambion, Grand Island, NY) and 5-mm stainless steel beads. The samples were macerated in a TissueLyser LT (Qiagen, Germantown, MD) for 2 min. miRNA was isolated using a mirVana Kit (Applied Biosystems, CA) in accordance with the manufacturer’s instructions, and the nucleic acid concentrations were calculated based on absorbance at 260/280 nM using a NanoDrop ND-1000 spectrophotometer (Thermo Scientific, West Palm Beach, FL). For each sample 200 ng of miRNA was reverse transcribed with the High Capacity cDNA Reverse Transcription kit and the TaqMan MicroRNA Reverse Transcription Kit (Applied Biosystems, CA).

Benign tissues obtained through transurethral resection of the prostate in six patients with benign enlargement were employed as controls.

### Ethics

Approval for the study was given by the Institutional Board of Ethics (CAPPesq – Comissão de Ética para Análise de Projetos de Pesquisa) under the numbers 054/13 and 352.893/2013.

## Results

### Efficacy of transfection

**Fig. 1:**
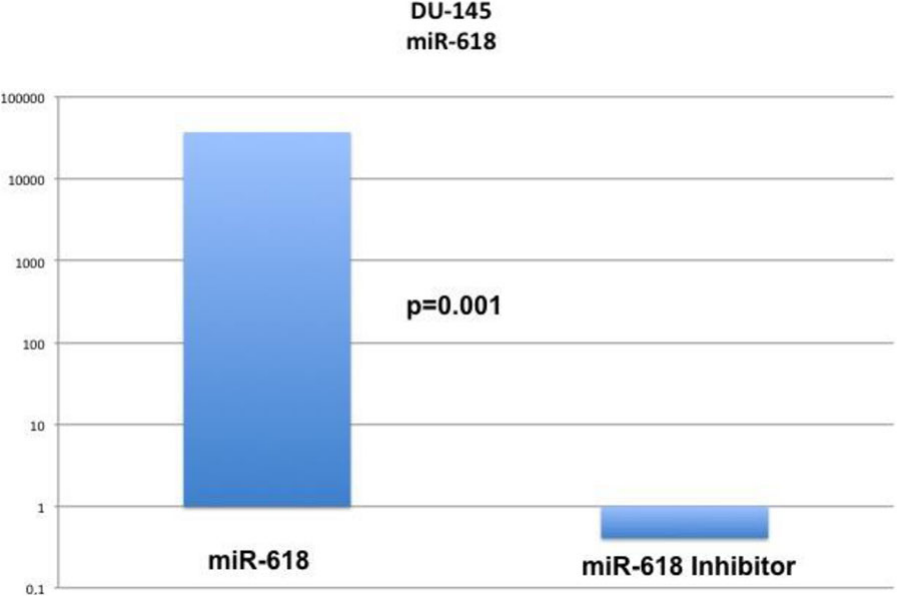
qRT-PCR confirms efficient transfection of miR-618 and its antagonist into DU145

Transfection success was evaluated with qRT-PCR, and relative expression was determined according to the 2^-ΔΔct^ method [7]. miR-618 and miR-618 inhibitor were both effectively transfected into DU-145 cells (Fig. 1).

### TIMP-1 and MMP-9 expression

**Fig. 2:**
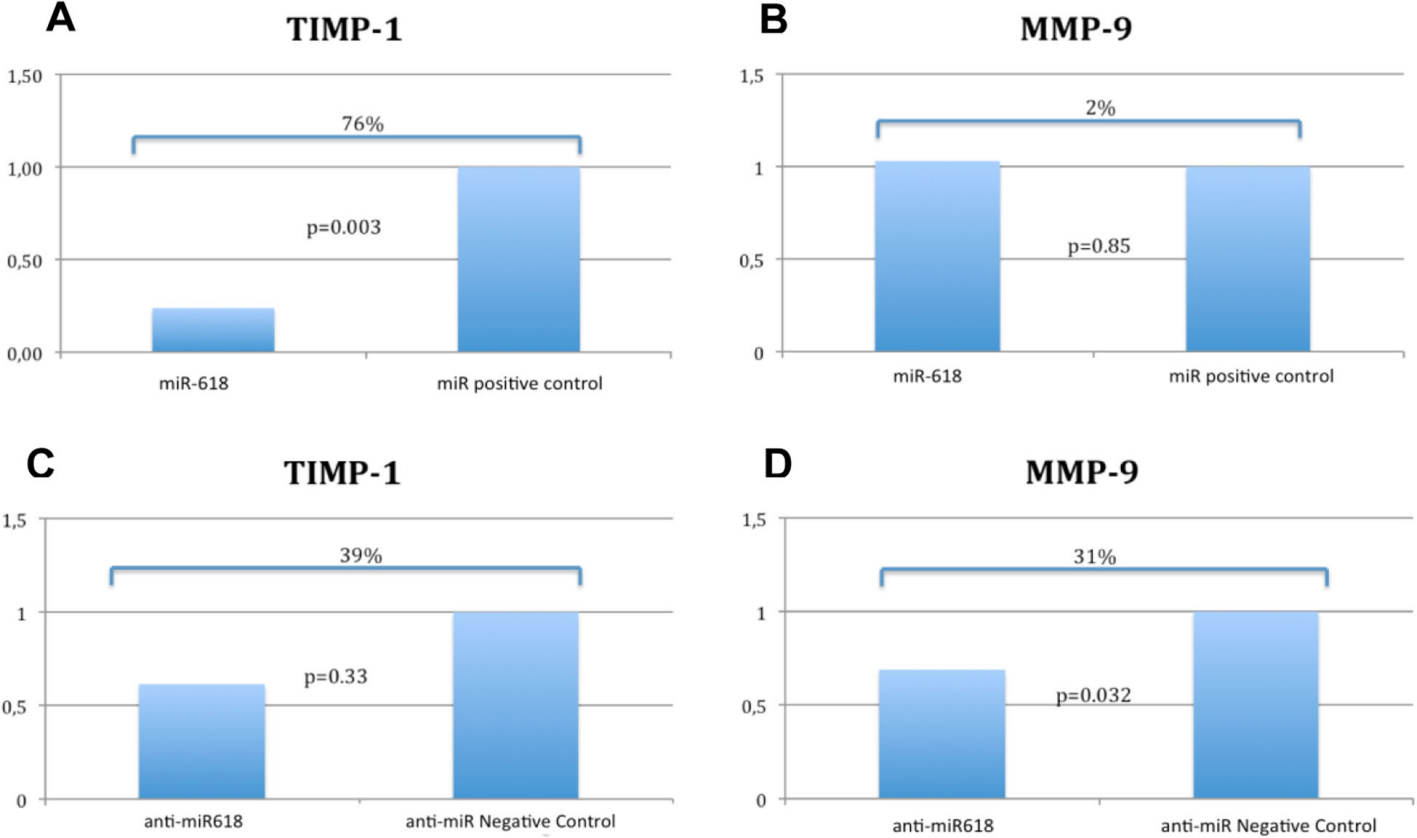
TIMP-1 and MMP-9 qRT-PCR expression in DU145 cells after transfection of miR-618, anti-miR-618 and their controls

Analysis of TIMP-1 and MMP-9 gene expression in DU145 cells after transfection of miR-618 or miR-618 inhibitor and in the corresponding positive and negative controls was performed. After miR-618 transfection, there was a 76% reduction of TIMP-1 levels relative to the corresponding control (*p* = 0.003), whereas MMP-9 expression did not differ (*p* = 0.85) (Fig. 2a and b). However, after anti-miR-618 transfection, TIMP-1 expression did not differ from the control level (*p* = 0.33), whereas MMP-9 was significantly reduced by 31% (*p* = 0.032) (Fig. 2c and d).

### Elisa

**Fig. 3:**
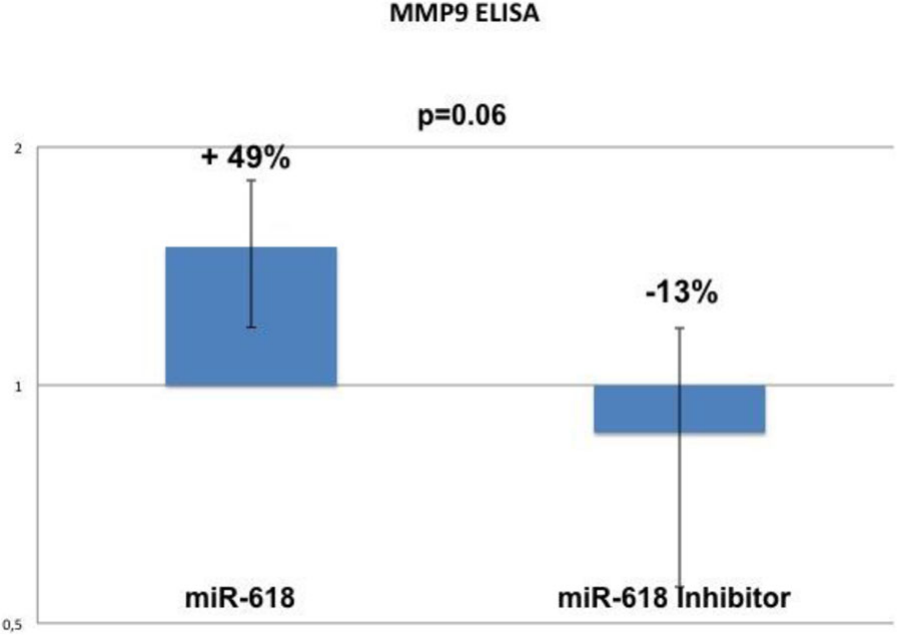
ELISA results obtained with DU145 confirms reduced levels of MMP-9 after miR-618 inhibition

The ELISA assay performed with the DU145 cells revealed a small reduction of MMP-9 levels after anti-miR-618 transfection (− 13%) when compared to miR-618 (+ 49%), and this reduction was almost statistically significant (*p* = 0.06) (Fig. 3).

### Zymography

The conditioned medium of anti-miR-618 transfected DU145 cells recovered from the top of the Matrigel membrane showed a 25% reduction of gelatin degradation relative to that observed in the conditioned medium of cells transfected with miR-618. However, this reduction did not achieve statistical significance (*p* = 0.55).

### miR-618 expression in radical prostatectomy specimens

Tissue samples of PCa obtained after radical prostatectomies performed by one of the authors (MS) were subjected to total miRNA extraction according to protocols described in Methods. A total of 51 patients were included in the analysis, and clinical data, i.e., PSA level, final pathological status, Gleason score and biochemical recurrence, were examined for correlations with miR-618 expression. The follow-up time was 61.7 months (SD, 41 months).

The mean expression of miR-618 for the whole cohort was 0.72. There was a progressive decrease in miR-618 expression from Gleason score < 7 (1.003) to Gleason score > 7 patients (0.41), and a marginally significant difference was found between subjects with Gleason score = 7 (0.79) and Gleason score > 7 (*p* = 0.08). In addition, the mean PSA levels were higher for the subjects with Gleason score > 7 (8.33 ng/dl) than for those with Gleason score = 7 (7.01 ng/dl, *p* = 0.01). Mean miR-618 expression was 0.64 for patients with positive biochemical recurrence and 0.54 for those who were negative (*p* = 0.69). miR-618 expression levels did not differ between patients with positive biochemical recurrence and those with negative recurrence when evaluating each Gleason score category separately.

Regarding final pathological status, miR-618 expression was lower in pT3 patients (0.48) than in pT2 patients (0.81) (*p* = 0.128). Patients with Gleason 7/pT2 had higher miR-618 expression (0.86) than did those with Gleason > 7/pT2 (0.35) (*p* = 0.07). The same trend was observed when comparing Gleason 7/pT3 (0.60) and Gleason > 7/pT3 subjects (0.46), although the difference was not significant (*p* = 0.47) (Table 1).

**Table 1:** Summary statistics of PCa samples and relative of expression of miR-618 (in parentheses) in PCa samples from 51 patients submitted to radical prostatectomy

		Total	Gleason score < 7	Gleason score = 7	Gleason score > 7	
Number of Patients		51 (0.72)	12 (1.003)	19 (0.79)	20 (0.41)	*p* = 0.08 ^(*)^
N^o^ Recurrence	Positive	14 (0.64)	1 (0.93)	7 (0.81)	6 (0.29)	*p* = 0.62
	Negative	25 (0.54)	7 (0.58)	10 (0.63)	12 (0.42)	
Mean PSA (ng/dl)		7.11	5.31	7.01	8.33	*p* = 0.01^(**)^
Pathological Status	N^o^ pT2	35 (0.81)	11 (1.08)	14 (0.86)	9 (0.35)	*p* = 0.07 ^(***)^
	N^o^ pT3	16 (0.48)	1 (0.14)	5 (0.60)	10 (0.42)	*p* = 0.4

* *p* = 0.08 Gleason score = 7 vs Gleason score > 7

** *p* = 0.01 Gleason score < 7 vs Gleason score > 7

***p = 0.07 pT2/Gleason score = 7 vs pT2/Gleason score > 7

## Discussion

The idea that metastasis reflects an imbalance between MMPs and TIMPs is not new. The concentration of MMPs is higher than that of TIMPs in malignant cell cultures and prostatic tissue [4]. Divergence in MMP and TIMP concentrations has also been identified in the blood of patients with malignant neoplasms of the prostate [5].

In situ hybridization studies have suggested that reduced TIMP-1 and TIMP-2 expression rather than increased MMP-2 and MMP-9 expression in PCa samples with high Gleason scores could be used as markers of disease severity [9]. Consistent with our findings, Still et al. [10] found that TIMP-2 expression was reduced in prostatic cancer samples, whereas MMP expression was increased [9, 11] . Our group previously demonstrated that in surgically removed prostatic tissue specimens, MMP-9 levels are high, whereas TIMP-1 levels are low [12].

miRNAs act by inhibiting mRNA at the posttranscriptional level. Some studies have suggested that miR-618 is overexpressed in neoplastic tissues. miR-618 overexpression was found to be present in 72% of patients with both hepatocellular carcinoma and hepatitis C virus [13], and a study that evaluated the expression of several miRNAs involved in the transition of Barrett’s esophagus to esophageal adenocarcinoma found overexpression in cancer tissue. One of the miRNAs was miR-618, for which increased expression was observed over the progression from normal tissue to esophageal cancer [14]. These findings indicate that as tissues acquire neoplastic characteristics, the amount of miR-618 increases, leading to reduced levels of TIMP-1. Reductions in TIMP-1 can be expected to release the activity of metalloproteinases, in particular MMP-9, thereby increasing the capacity for cell invasion.

Publications addressing TIMP-1 control by miRNAs in oncology are scarce. Lu et al. [15] found that TIMP-1 can be downregulated by miR-196 in oral cancer cells, and other researchers have reported that miR-1293 is able to control TIMP-1 in 293 T cells [16]. However, none of those miRNAs have been reported to have roles in PCa. Our study sought to address whether miR-618, which has a binding site at the 3’-UTR of TIMP-1 mRNA, can regulate the expression of TIMP-1. Song et al. reported that miR-618 overexpression inhibits prostate cancer-cell invasion and migration not through TIMP-1 or MMP-9 regulation but through the FOXP2 gene [7]. However, in contrast to these results, the present study suggests that miR-618 may act as an oncomir: When overexpressed in DU145 cells, the relative expression of TIMP-1 is markedly reduced, whereas MMP-9 expression is unaffected. However, after miR-618 inhibitor transfection, TIMP-1 levels, although lower than in controls, did not significantly differ from control levels. These levels corresponded to an approximately three-fold increase in TIMP-1 levels relative to those obtained after miR-618 transfection. As a consequence of increased TIMP-1 expression, we observed downregulation of the MMP-9 gene. This result was corroborated by the ELISA assay, which demonstrated that MMP-9 levels were higher after miR-618 overexpression. Thus, the findings of this qRT-PCR study with the DU145 cell line suggests links between miR-618 and both TIMP-1 and MMP-9.

After the in vitro study, we evaluated miR-618 expression in PCa samples. We expected results similar to those reported by Abdalla and Haj-Ahmad [13] for hepatocellular carcinoma and by Wu et al. [14] for esophageal cancer. However, unexpectedly, the expression levels of miR-618 in our PCa samples were lower for cases with higher Gleason scores and higher pathological status. This suggests that miR-618 either acts as a tumor suppressor or is only a biological marker of disease severity. This dual behavior might reflect a limitation of the present study as the in vitro experiments were performed with DU145, which is an androgen-independent cell line, whereas the patient cohort had localized prostate cancer. Another possibility is that miR-618 is a context-dependent miRNA: For the in vitro studies, we used highly aggressive PCa cells, and miR-618 appeared to be an oncomiR; however, the miR-618 levels of surgical specimens from patients with clinically localized disease suggest that miR-618 acts as a tumor-suppressor miR. An example of such behavior is found in miR-100: It may be an oncomiR, as it controls cell cycle-related genes; but it may also function as a tumor suppressor miR [17].

## Conclusions

miR-618 can regulate TIMP-1 expression, which may indirectly affect MMP-9 expression in vitro. These effects may play a role in the metastatic potential of prostate cancer cells. However, the increased expression of miR-618 in samples from patients with PCa suggests a protective role against more aggressive disease. More detailed studies are needed to determine whether miR-618 directly controls the TIMP-1 molecule or whether it acts as a context-dependent miRNA. Until this uncertainty is resolved, we cannot develop therapies aimed at changing miR-618 levels in PCa.

## Abbreviations

miR: MicroRNA
MMP: Matrix metalloproteinase
PCa: Prostate cancer
qRT-PCR: Quantitative real-time polymerase chain reaction
TIMP: Tissue inhibitor of metalloproteinases
